# Dyslipidemia in rheumatoid arthritis: the possible mechanisms

**DOI:** 10.3389/fimmu.2023.1254753

**Published:** 2023-10-25

**Authors:** Jiahui Yan, Sisi Yang, Liang Han, Xin Ba, Pan Shen, Weiji Lin, Tingting Li, Ruiyuan Zhang, Ying Huang, Yao Huang, Kai Qin, Yu Wang, Shenghao Tu, Zhe Chen

**Affiliations:** ^1^ Department of Integrated Traditional Chinese and Western Medicine, Tongji Hospital of Tongji Medical College of Huazhong University of Science and Technology, Wuhan, China; ^2^ Department of Geriatrics, Tongji Hospital of Tongji Medical College of Huazhong University of Science and Technology, Wuhan, China; ^3^ Department of Rheumatology and Immunology, Zhongnan Hospital of Wuhan University, Wuhan, China

**Keywords:** dyslipidemia, rheumatoid arthritis, cardiovascular disease, high-density lipoprotein cholesterol, low-density lipoprotein cholesterol, mechanism

## Abstract

Rheumatoid arthritis (RA) is an autoimmune inflammatory disease, of which the leading cause of death is cardiovascular disease (CVD). The levels of total cholesterol (TC), low-density lipoprotein cholesterol (LDL-c), and high-density lipoprotein cholesterol (HDL-c) in RA decrease especially under hyperinflammatory conditions. It is conflictive with the increased risk of CVD in RA, which is called “lipid paradox”. The systemic inflammation may explain this apparent contradiction. The increased systemic proinflammatory cytokines in RA mainly include interleukin-6(IL-6)、interleukin-1(IL-1)and tumor necrosis factor alpha(TNF-α). The inflammation of RA cause changes in the subcomponents and structure of HDL particles, leading to a weakened anti-atherosclerosis function and promoting LDL oxidation and plaque formation. Dysfunctional HDL can further worsen the abnormalities of LDL metabolism, increasing the risk of cardiovascular disease. However, the specific mechanisms underlying lipid changes in RA and increased CVD risk remain unclear. Therefore, this article comprehensively integrates the latest existing literature to describe the unique lipid profile of RA, explore the mechanisms of lipid changes, and investigate the impact of lipid changes on cardiovascular disease.

## 1 Introduction

Rheumatoid Arthritis (RA) is a systemic, inflammatory and autoimmune disease with an unknown cause. It involves predominantly synovial joints, leading to joint destruction and permanent disability (1, 2). There is a substantial burden on society and individuals associated with RA, which is estimated to occur globally at 0.46 percent (1, 3). Cardiovascular disease (CVD) is the primary cause of death in RA, one of the most prevalent chronic inflammatory diseases. (1, 4). In RA patients, CVD is more prevalent than in the general population, and it is equal to the prevalence of type 2 diabetes (5). Therefore, the AHA/ACC guidelines of the American Heart Association and the American Heart Association recommend that coronary artery calcium (CAC) be considered to evaluate subclinical atherosclerosis in patients with chronic inflammatory diseases, including RA patients (6).

It is well known that the traditional CVD risk factors include age, systemic hypertension, obesity, hypercholesterolemia, diabetes mellitus, smoking etc. (7). Generally, the atherogenic lipid profile, presented as elevated low-density lipoprotein cholesterol (LDL-c), total cholesterol (TC) and total glyceride (TG), decreased high-density lipoprotein cholesterol (HDL-c), can increase the risk of CVD. However, the risk of CVD in RA patients has been shown to be higher, accompanied with lower blood lipid levels (decreased LDL-c, TC and HDL-c). This contradictory phenomenon is known as the “lipid paradox” (8). Therefore, traditional risk factors cannot fully explain lipid paradox in RA (9). RA is an independent CVD risk factor confirmed by European Society of Cardiology Guidelines, and therefore the European League Against Rheumatism recommends that CVD risk assessment for RA patients should be performed at least every 5 years, and a 1.5 coefficient should be applied to the CVD risk score (10). Non-traditional risk factors, including systemic chronic low-grade inflammation related to RA, endothelial dysfunction, and genetics etc., may help explain the increased CVD risk of RA (7, 11–13). Therein, systemic chronic low-grade inflammation contributes to altered lipid profile and an increased risk of CVD in RA patients.

The development of RA involves various inflammatory cytokines and aberrant signaling pathway transductions, such as TNF-α, IL-6, IL-1, IL-10, growth hormone, granulocyte-macrophage colony-stimulating factor (GM-CSF), transforming growth factor β (TGF-β), interferon (IFN), fibroblast growth factor-2 (FGF-2), and others. IL-6 and IFN-γ primarily activate the Janus kinase/signal transducer and activator of transcription (JAK/STAT) pathway, IL-1β mainly activates the mitogen-activated protein kinase (MAPK) pathway, while TNF-α can simultaneously activate both JAK/STAT and MAPK pathways (14). Additionally, IL-1, TNF-α, and IL-6 are closely associated with the upregulation of the NF-κB pathway in RA patients (15). Although these inflammatory mediators are characteristic of RA, they also represent common inflammatory pathways in many other diseases. Among them, TNF-α, IL-1, IL-6, and the JAK/STAT pathway are the core mechanisms shared by RA and CVD (16, 17). The inflammatory cytokines in the local joints of RA can enter the circulation and trigger systemic inflammation. This further leads to lipid metabolism abnormalities, increased CVD risk, and mortality in RA patients (18). Assessing RA CVD risk through lipid evaluation may not be sufficiently objective, especially in the presence of lipid paradox. The phenomenon of lipid paradox in RA has attracted widespread attention. Therefore, it is crucial to find out how inflammation increases RA CVD risk through blood lipids.

## 2 Lipids and lipoproteins metabolism

**Figure 1:**
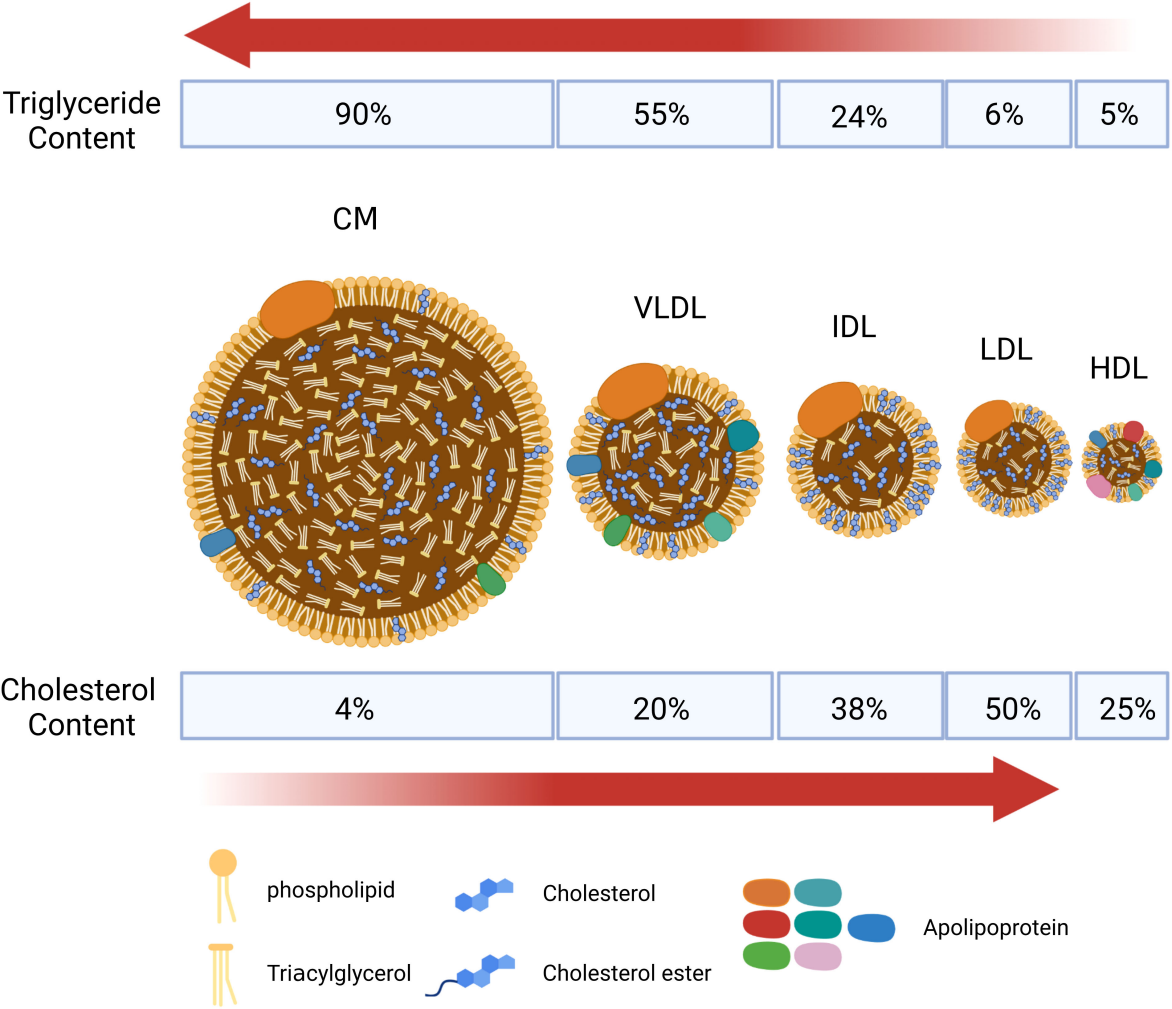
Schematic diagram of the structure of main lipid particles. Lipoproteins are complex particles with a hydrophobic core of non-polar lipids, mainly cholesterol esters and total glyceride (TGs). The hydrophobic core is surrounded by a hydrophilic membrane composed of phospholipids, free cholesterol, and apolipoprotein. According to the particle size, lipid composition, and apolipoprotein, lipoproteins are divided into chylomicrons (CM), chylomicron remnants, very low-density lipoprotein (VLDL), intermediate-density lipoprotein (IDL), low-density lipoprotein (LDL), high-density lipoprotein (HDL), and lipoprotein(a) [L p(a)]. CM and VLDL are rich in triglycerides, and LDL is rich in cholesterol.

**Figure 2:**
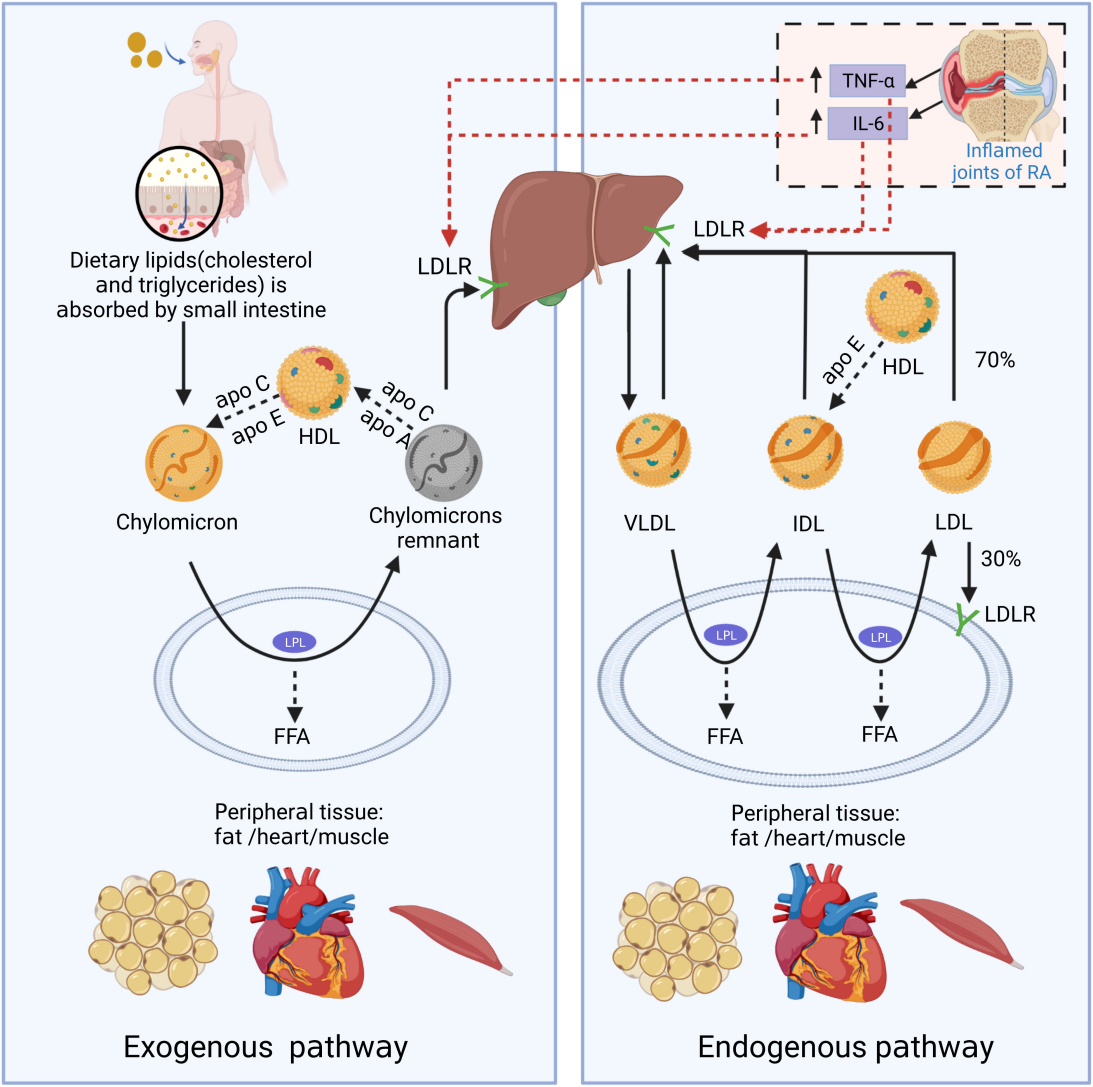
The schematic diagram of endogenous and exogenous cholesterol pathways. In the exogenous regulation pathway, lipids (cholesterol and triglycerides) from food sources are transferred to CMs through being absorbed into the blood in the small intestine. CMs reach peripheral tissues with the blood and exchange lipoproteins with HDL to obtain apolipoprotein E (Apo E). And CMs is metabolized by lipoprotein lipase (LPL) in fat and muscle cells to generate free fatty acids (FFAs) and chylomicron remnants. FFAs are absorbed by adjacent muscle and fat cells for energy production or storage. As the size of chylomicron decreases, the phospholipids and carrier proteins (Apo A and C) on the surface of the chylomicron are transferred to other lipoproteins (primarily HDL). Apo E on chylomicron remnants binds to LDL receptor (LDLR) and other liver receptors (such as low-density lipoprotein receptor-related protein 1 and syndecan-4), and is absorbed and cleared by liver cells. In the endogenous lipoprotein pathway, VLDL is produced by the liver, and enters the blood. TGs in VLDL is metabolized by LPL in peripheral tissues and generate FFAs and IDL. Those IDL particles are relatively enriched in cholesterol esters and obtain Apo E from HDL particles. In a pathway similar to the removal of chylomicron remnants, a small portion (about 50%) of the IDL particles can be removed from circulation by the liver through binding with Apo E and liver receptors, such as LDL and LRP receptors. The remaining TGs in IDL continue to release FFAs by LPL action, and the exchangeable carrier proteins are transferred from IDL particles to other lipoproteins, generating low-density lipoprotein (LDL). About 70% of circulating LDL is cleared by liver cell LDLR-mediated endocytosis, and the rest is absorbed by extra-liver tissues. In the state of RA inflammation, tumor necrosis factor-α (TNF-α) and interleukin-6 (IL-6) produced locally in the joints can enter the circulation. TNF-α and IL-6 can promote LDL metabolism by increasing the expression of LDLR on the surface of liver cells.

**Figure 3:**
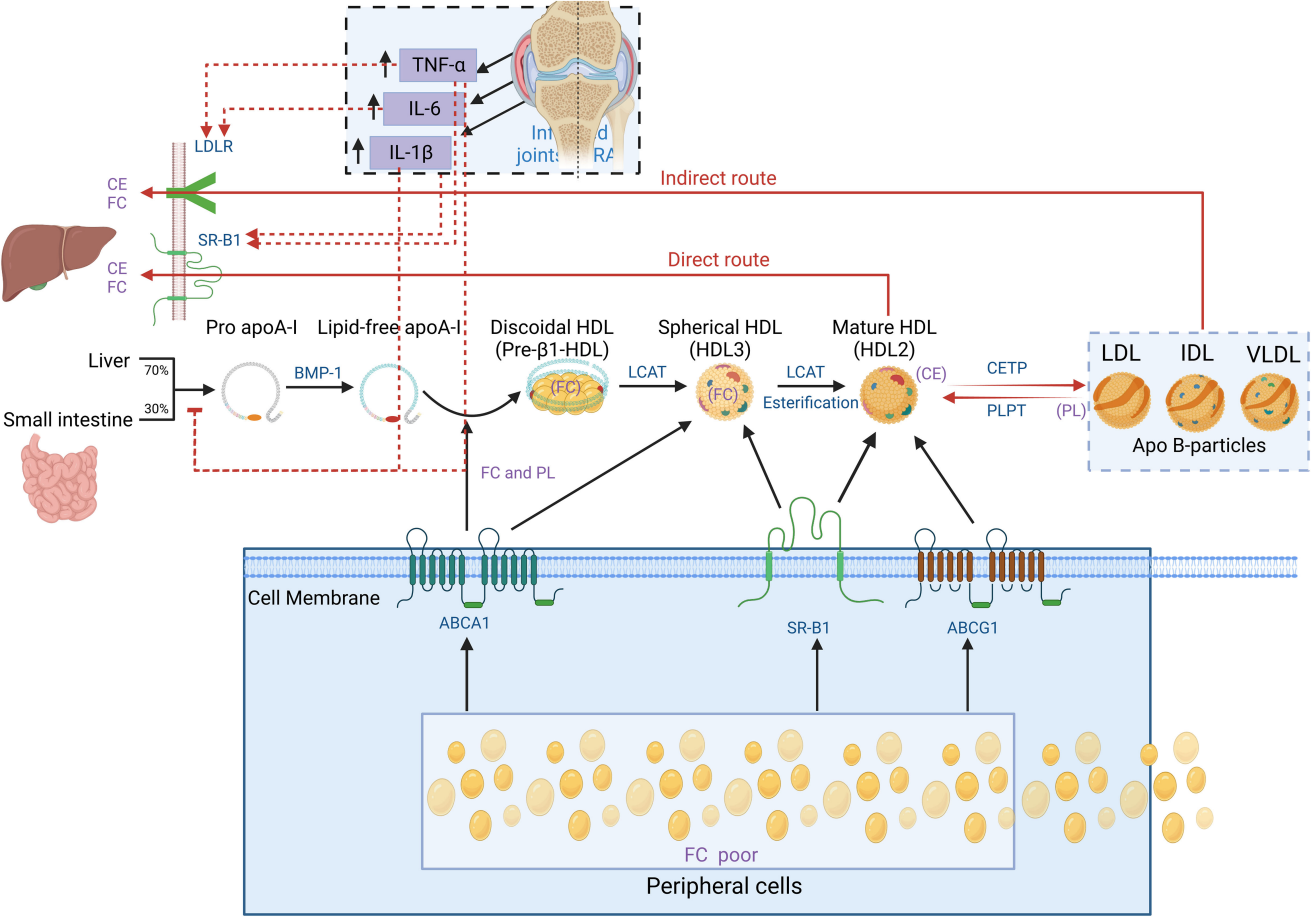
Schematic diagram of cholesterol reverse transportation. Reverse cholesterol transport (RCT) is a process that starts with the formation of pro-Apo A-I in the liver and intestines. Bone Morphogenetic Protein-l (BMP-1) converts it into mature Apo A-I. Free cholesterol (FC) from peripheral cells (including macrophages) flows out to the lipid-free Apo A-I by interacting with ATP Binding Cassette Subfamily A Member l (ABCA1), forming disc-shaped nascent HDL particles. The above process is called cholesterol efflux. Apo A-I on the disc-shaped HDL particle directly interacts with Lecithin Cholesterol Acyltransferase (LCAT) synthesized by the liver to convert the disc-shaped HDL into spheroidal HDL particles. Then, LCAT converts cholesterol into Cholesterol Ester (CE), resulting in cholesterol esterification and HDL maturation. Mature HDL can also obtain additional cholesterol from cells through enzyme ATP-binding cassette transporter G1 (ABCG1) and scavenger receptor B1 (SR-B1). In the direct pathway, the HDL particle docks with SR-B1, which regulates the transfer of cholesterol from HDL particles to cells. In the indirect pathway, the cholesterol transferred by HDL particles is transferred to lipoproteins containing Apo B (e.g., LDL-c and VLDL-c) through cholesterol ester transfer protein (CETP). At the same time, phospholipid transfer protein (PLTP) transfers phospholipid (PL) from lipoproteins containing Apo B to HDL. Finally, both the direct and indirect RCT pathways result in the transfer of cholesterol from peripheral sites (mostly macrophages) to the liver and excretion through bile. In the state of RA inflammation, tumor necrosis factor-α (TNF-α), interleukin-6 (IL-6) and interleukin-1β (IL-1β) produced locally in the joints can enter the circulation. TNF-α and IL-6 can promote LDL metabolism by increasing the expression of LDLR and SR-B1 on the surface of liver cells. TNF-α and IL-1β can inhibit the production of pro-Apo A-I particles in the liver, suppressing HDL generation. As a result, levels of both HDL and LDL in RA decrease.

It is essential to understand the basic process of lipid metabolism in order to understand the significance of lipid abnormalities in RA. In general, the majority of lipids (cholesterol and TGs) cannot dissolve in water, and their transportation in the bloodstream relies on lipoproteins. According to the particle size, lipid composition, and apolipoprotein, lipoproteins are divided into chylomicrons (CM), chylomicron remnants, very low-density lipoprotein (VLDL), intermediate-density lipoprotein (IDL), low-density lipoprotein (LDL), high-density lipoprotein (HDL), and lipoprotein(a) [L p(a)] (**Figure 1**). Among them, chylomicron remnants, VLDL, IDL, LDL, and L p(a) are pro-atherosclerotic, while HDL is anti-atherosclerotic. Blood lipids are transported from the small intestine to the liver and peripheral tissues via endogenous and exogenous pathways (**Figure 2**) (19). In the endogenous pathway, the levels of low-density lipoprotein receptor (LDLR) in the liver are important factors for regulating plasma LDL levels. For instance, increased liver LDLR levels can increase LDL clearance rate, resulting in lower plasma LDL levels (20). There is a reverse transport mechanism in the body that transfers excess lipids from peripheral tissues to the liver, which is called reverse cholesterol transport (RCT) (19). ATP-binding cassette subfamily A member 1 (ABCA1), Lecithin cholesterol acyltransferase (LCAT), ATP-binding cassette transporter G1 (ABCG1), scavenger receptor class B type I (SR-B1) and cholesteryl ester transfer protein (CETP) play key roles in the process of RCT (**Figure 3**).

## 3 The lipid profile in RA

Researches show that before the diagnosis of RA patients (even up to 10 years prior), their blood lipids change already (21, 22). The changed lipid parameters include TC, TG, HDL, LDL, L p(a), Apo A-I, Apo B, TC/HDL-c and LDL-c/HDL-c etc. The results of those related studies are not entirely consistent (**Supplementary Table 1**, see the **Supplementary Material**). It is generally acknowledged that the lipid profile of preclinical and early RA patients is similar, showing mild atherosclerotic characteristics due to systemic low-grade inflammation and metabolic syndrome (21, 23–26). It presents as normal or mildly elevated TC, LDL-C, and TG, which is related to the decreased HDL-c level. In contrast, patients with established progressive RA have long-term or recurrent high levels of inflammation. The process of recurrence and remission can lead to lipolysis and decreased levels of lipid (mainly TC and LDL), which refers to “lipid paradox” (18, 22, 26–30). The consistent finding is that the HDL levels of RA patients decrease even in different disease states (31). However, some studies have shown different results, such as significant decreases in TC and LDL levels but a rise in HDL levels than the general population in female RA individuals, showing an inhibitory pattern of atherosclerosis (32). This indicates that abnormal blood lipids in RA may also be related to factors other than inflammation, such as gender, hormones, and genotype.

The reasons for the inconsistent studies about blood lipid levels in RA are mainly involved in following factors (33, 34): (1) confounding effects of differences in age, obesity level, smoking, and gender between RA patients and control groups; (2) a small sample size resulting in lack of reliability; (3) significant disease-related heterogeneity among the studies, such as early-stage *vs*. late-stage disease, active *vs*. inactive disease, and menopausal *vs*. non-menopausal patients; (4) confounding effects of steroid and other drug treatments. For better-designed studies in the future, it may be helpful to summarize these reasons.

Studies have shown that the altered subfractions and functions of blood lipids also occur when the levels of RA lipids change in an inflammatory state. Lipid metabolism abnormalities can further exacerbate autoimmunity, inflammation, and CVD risks in RA, creating a vicious cycle (35). Therefore, analyzing the mechanisms of lipid metabolism disorders in RA and attempting to intervene will help break this vicious cycle. The following analysis primarily focuses on the changes in quantitative and qualitative aspects of lipoprotein particles in RA blood lipid metabolism, and attempts to explain their correlation with increased CVD risks.

## 4 The subfractions and function of HDL change in RA

As HDL is an innate immune system component, HDL function is related to inflammation status. In exchange for increased host defense, HDL’s metabolic functions are sacrificed (36). In metabolic syndrome associated with chronic low-grade inflammation, HDL may have a high degree of functional impairment. Similarly, RA patients, especially in the active phase, exhibit significant abnormalities in HDL, which can be summarized as a reduction in anti-atherosclerotic subfractions (large HDL2 particles) and a shift in HDL function from anti-inflammatory to pro-inflammatory. The mechanism of pro-inflammatory HDL formation may involve the changes of key proteins and lipids. Finally, based on the evidence collected, attempts were made to speculate on the mechanisms of HDL decline in RA.

### 4.1 The level of anti-atherosclerotic HDL2 decreases in RA

The functions of HDL depend on its subfraction partially. The subfractions of HDL includes small particles pre-β-HDL, HDL3, and large particles HDL2. HDL matures through the gradual transition of pre-β-HDL→HDL3→HDL2. It is known that high levels of HDL2-chol are associated with the anti-atherosclerotic effect of HDL (37). Studies have shown that the concentrations of HDL3-chol and HDL2-chol (especially HDL2-chol) in RA are significantly decreased and appear to be associated with disease activity (38–40). One possible mechanism for that is the inflammation in RA inhibits HDL’s maturation process. By catalyzing the esterification of free cholesterol within HDL, LCAT promotes the conversion of new spherical or disc-shaped HDL3 into mature spherical HDL2 (**Figure 3**). In the inflammatory state, cytokines such as TNF and TGFβ may impair the activity of LCAT, thus inhibiting HDL maturation and leading to an increase in small pre-β-HDL particles and a decreased large HDL2 particles (41–43). In addition, Apolipoprotein A-I (Apo A-I) in HDL is the most important physiological activator of LCAT and produces an important effect on the catalytic function of LCAT. For example, LCAT lacks the efficiency to mediate cholesterol esterification in lipoproteins, but its activity is significantly enhanced several orders of magnitude in the presence of Apo A-I (44). Therefore, the reduced level of Apo A-I in RA is believed to reduce the activity of LCAT in catalyzing HDL maturation. As a result, the decreased of HDL2 may weaken the ability of HDL to resist atherosclerosis in RA.

### 4.2 The anti-inflammatory HDL in RA becomes pro-inflammatory

Under normal conditions, HDL assumes responsibility for inhibiting LDL oxidation and promoting cholesterol efflux from vascular foam cells (19, 45). It is a protective “anti-inflammatory HDL” with anti-atherosclerotic properties. Long-established studies have shown that HDL-C levels are negatively correlated with cardiovascular disease risk (46). The use of drug interventions to increase HDL-C levels, however, has not provided cardiac protection for high-risk populations in several large trials (47). Multiple studies have shown that the anti-inflammatory property of HDL is impaired during inflammation in animals and humans, and HDL loses the ability to remove cholesterol from atherosclerotic plaques and protect LDL from oxidation, becoming pro-inflammatory HDL (48–55). That causes atherosclerosis and increases CVD risk.

Similarly, studies have shown that RA patients have higher pi-HDL levels than healthy controls (36). The anti-inflammatory and antioxidant functions of pi-HDL in RA are also abnormal, which is associated with disease activity (56). This may explain that the fundamental reason for the increased CVD risk in RA may be changes in the properties and functions of HDL under inflammatory conditions, whereby anti-inflammatory HDL becomes pro-inflammatory HDL. As a result, HDL in RA have the less ability to perform an RCT, anti-inflammatory, and antioxidant function (56).

### 4.3 The changes of HDL-associated proteins correlate with the formation of pi-HDL in RA

**Figure 4:**
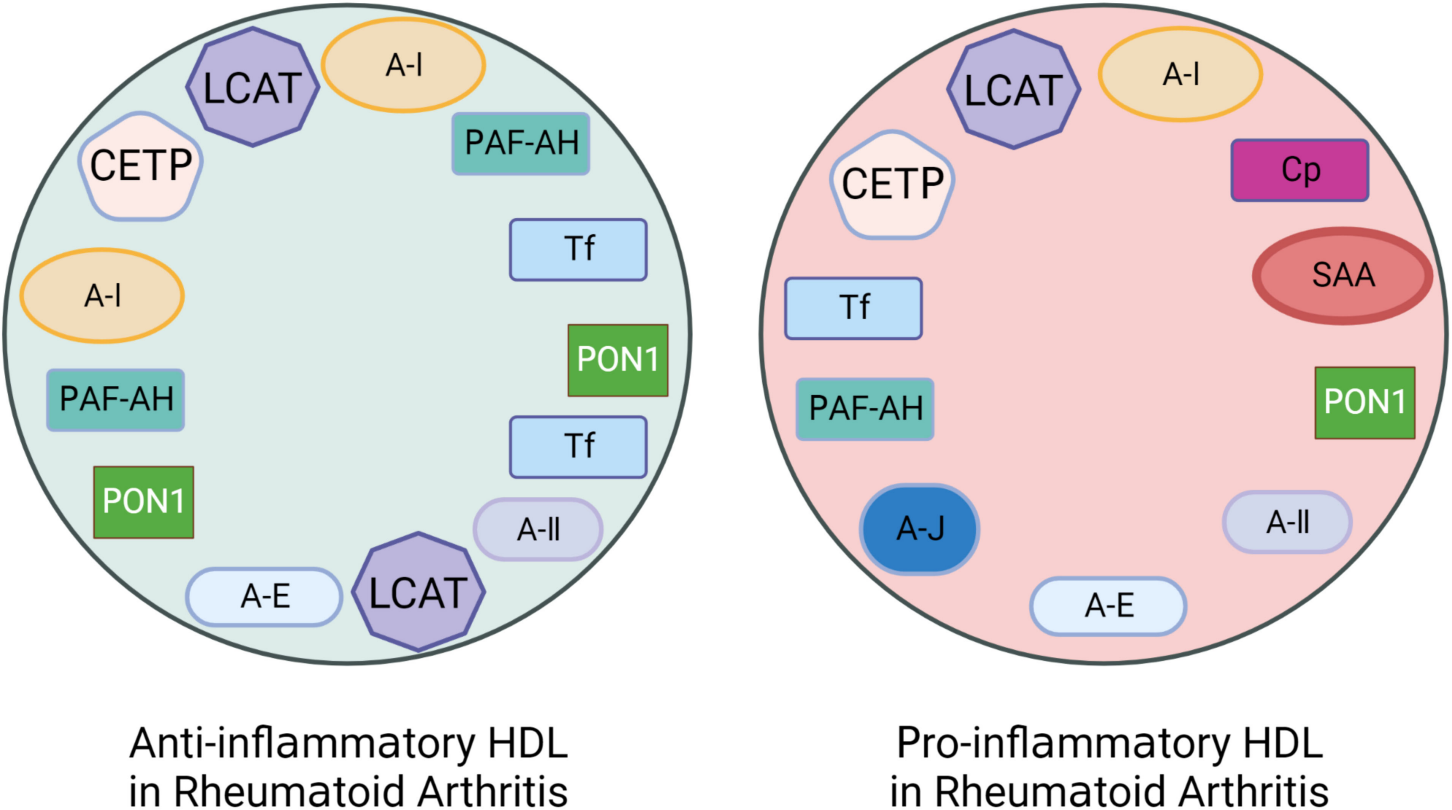
The altered protein components of pro-inflammatory HDL in patients with Rheumatoid Arthritis (RA). Rheumatoid arthritis (RA) patients have lower levels of anti-inflammatory and antioxidant proteins that are associated with HDL’s anti-inflammatory functionality. These proteins include Apolipoprotein A-I (Apo A-I), Paraoxonase-1 (PON-1), Platelet-activating Factor Acetyl Hydrolase (PAF-AH), LCAT, CETP, and Apo J etc. However, the levels of pro-inflammatory proteins such as Serum amyloid A (SAA), Ceruloplasmin (Cp), Transferrin (Tf), and myeloperoxidase (MPO) are increased.

It is known that the function of blood lipids mainly depends on their protein composition (19). Each HDL particle carries Apo A-I and may also carry other lipoproteins, such as Apo A-II, Apo A-IV, Apo E, Apo C. More than 85 different functional accessory proteins temporarily bind to HDL particles, including LCAT, CETP, platelet activating factor-acetyl hydrolase (PAF-AH), serum amyloid A(SAA) and paraoxonase1(PON-1) etc. (57–59). One of the reasons for the formation of dysfunctional pi-HDL may be changes in the HDL protein composition. Studies have shown that anti-inflammatory and antioxidant factors are reduced while pro-inflammatory proteins are increased in pi-HDL protein particles (**Figure 4**).

In HDL, Apo A-I is the main functional and structural protein, representing about 70% of the total protein. (60). Plasma Apo A-I is involved in cholesterol reverse transport and anti-inflammatory process, which can delay the progression of atherosclerosis (61, 62). Apo A-I is also a framework for HDL to bind to other proteins, and interacts with cell transport proteins to promote the transfer of lipids to HDL (47). Therefore, changes in the level or properties of Apo A-I may greatly affect the function of HDL (63). On the one hand, studies have found that the serum level of Apo A-I in RA patients is significantly reduced. It may be related to the inhibition of Apo A-I production in the liver, possibly through the kinase c-jun-N-terminal kinase (JNK) signaling pathway activated by the inflammatory cytokine TNF-α and the promotion of secretory phospholipase A2 (sPLA2)-mediated Apo A-I degradation during the inflammatory acute phase response, which may be related to inflammation inhibiting liver production of Apo A-I and promoting its decomposition process (64–67). On the other hand, posttranslational modifications of RA Apo A-I also lead to abnormal HDL function. Levels of myeloperoxidase (MPO) in RA patients significantly increase, and the MPO-specific 3-chlorotyrosine and 3-nitrotyrosine content on HDL also significantly increase. Meanwhile, the cholesterol efflux capacity of RA decreases (68). That is similar to coronary heart disease patients. It is known that MPO can target Apo A-I in plasma and arterial lesions, and result in nitration and chlorination of specific tyrosine residues in Apo A-I, which is associated with the loss of specificity of cholesterol receptor activity mediated by ABCA1 (69–72). Therefore, the function of HDL in the RCT process is suppressed.

The antioxidant activity of HDL is mainly attributed to paraoxonase-1 (PON-1) and platelet-activating factor acetyl-hydrolase (PAF-AH) located on its surface (48, 73). PON-1 is synthesized in the liver, secreted into the blood and combine with HDL (74). PON-1 can prevent LDL oxidation and has important anti-inflammatory effects, responsible for the anti-atherosclerotic properties of HDL (48, 75, 76). Studies have shown that the serum PON-1 activity in coronary heart disease patients and RA patients is decreased, which is related to chronic inflammation and impaired antioxidant defense (40, 56, 77, 78). Possible mechanisms include increased reactive oxygen species (ROS) consumption of PON-1 in HDL, reduced hepatic production of PON-1 or affected active sites of PON-1under RA inflammatory conditions (79, 80). However, after the use of TNF-α inhibitors to treat RA, HDL antioxidant capacity significantly improves, and stable increases in PON-1 activity are observed (81). Therefore, it is speculated that the decrease in PON-1 activity/level under RA inflammation leads to impaired antioxidant function in HDL.

Platelet-activating factor (PAF) is a type of bioactive lipid that promotes inflammation. PAF can be hydrolyzed and inactivated by PAF-AH. The level of PAF-AH in the plasma of RA patients is significantly decreased (81–83). Similarly, during the acute inflammatory response, PAF-AH in HDL decreases significantly(48). Therefore, it can be reasonably speculated that the inflammatory state in RA reduces the antioxidant capacity of HDL by reducing the activity of PAF-AH and PON-1 on HDL.

As a highly sensitive acute-phase reactant, serum amyloid A (SAA) is linked to a number of chronic inflammatory diseases, including obesity, coronary heart disease, and cancer. (84–86). SAA is an important component of lipoproteins and HDL (87). When inflammation is severe, SAA can be the major apolipoprotein of HDL (86). Studies have shown that the average concentration of SAA in most RA patients is significantly higher than normal levels, and it is positively correlated with the level of inflammatory cytokines, such as ESR, CRP, IL-4, IL-6, IL-10, IL-17, rheumatoid factor (RF) and anti-cyclic citrullinated peptide antibody (ACCP)(88). The level of SAA in acute-phase HDL increases, accompanied by the loss of Apo A-I (48). HDL particles containing Apo A-I appear to contain most of the LCAT, which is responsible for the esterification of plasma cholesterol and is the HDL ligand responsible for interaction with cells (89). Substituting Apo A-I with SAA may inhibit LCAT activity in HDL, inhibit cholesterol efflux from peripheral cells, and relocate HDL and its lipids to other targets (48). Compared with unmodified HDL structure, the HDL/SAA structure binds more readily with macrophages and less with liver cells. This may be related that when there is inflammation, HDL/SAA binding sites increase on macrophages and decrease on liver cells. (90). This suggests that similar to the acute phase, RA serum SAA may replace Apo A-I particles in HDL, inhibit the cholesterol efflux capacity of HDL by inhibiting LCAT activity, and enhance the binding of HDL/SAA to macrophages.

Lecithin-cholesterol acyltransferase (LCAT) is a key enzyme that catalyzes the esterification of plasma free cholesterol, mainly expressed in the liver. As mentioned earlier, LCAT catalyzes the esterification of HDL’s free cholesterol, which helps HDL mature and also promotes the acceptance of excess cholesterol from macrophages (the first step of RCT). Studies have shown that inflammatory factors (TNF and TGF-β) can impair LCAT activity and reduce the role of HDL-mediated RCT. The degree of impairment of RCT by inhibiting LCAT activity is almost the same as that under inflammatory conditions (43, 91). It has been found that the activity of serum LCAT in RA patients is significantly decreased (36, 92). The decrease in RA LCAT activity may be partially attributed to the decrease in the concentration of Apo A-I (known as LCAT activity co-factor), and may also be related to the inhibition of LCAT activity and expression levels by inflammatory cytokines and ox-LDL (41). Overall, RA inflammation may lead to a decreased LCAT activity and level, leading to inhibit the maturation process of HDL, and weaken the RCT function of HDL.

Cholesteryl ester transfer protein (CETP) can participate in RCT process, where CETP transfer the cholesterol ester of HDL to lipoproteins containing apolipoprotein B, and further transfer it to the liver through the cholesterol ester uptake process mediated by LDLR (93). Studies have shown that the level and activity of CETP in RA patients are significantly reduced, and are related to the increased CVD risk in RA (94, 95). The mechanism may involve that the CETP activity is inhibited by inflammation of RA. As a result, the ability to transfer excess cholesterol from HDL to VLDL, LDL, and CM particles and CETP-mediated RCT process are inhibited. However, there are also studies showing that CETP activity is increased in RA patients, which may be one of the reasons for HDL dysfunction (40, 96). This has been found consistently in patients with metabolic syndrome and diabetes. The CETP inhibitor torcetrapib inhibits CETP activity, increases HDL levels, but increases blood pressure and cardiovascular events. Inhibiting CETP does not necessarily mean improving the function of HDL despite increasing its levels (97). Therefore, more researches may be needed to fully understand the changes of CETP level/activity in RA and their overall impact on RA-related CVD.

Ceruloplasmin (Cp) is a plasma glycoprotein mainly synthesized and secreted by the liver into the bloodstream (98). Cp is a protein associated with HDL (99). In the body, Cp is able to oxidize Fe^2+^ to Fe^3+^, and transfers Fe^3+^ to transferrin (Tf) for transport in the plasma, thereby reducing the concentration of free Fe^2+^ (which can cause Fe^2+^-catalyzed lipid peroxidation) and producing an effective antioxidant effect (100). Studies have found that the serum Cp concentration in RA patients is significantly increased and the transferrin level is decreased, and there is a statistically significant positive correlation between Cp serum concentration and ESR (101). Cp is an acute-phase reactant, as is known (102, 103). During inflammation or infection, Cp significantly increases, mainly due to pro-inflammatory IL (such as IL-1 and IL-6) stimulating the production of Cp by liver cells (104). The increased serum Cp concentration may be explained by the inflammation accompanying RA disease. However, studies have clearly shown that serum Cp is an independent risk factor for CVD (105). Cp may play an important role in atherosclerosis and plaque rupture by mediating the oxidative modification of LDL in smooth muscle cells and endothelial cells, as well as the formation of ox-LDL (106, 107). Therefore, the increase in Cp level caused by RA inflammation may be an adverse indicator of the RA CVD risk.

Transferrin (Tf) is a metal-binding protein that is associated with HDL, and can prevent LDL oxidation (108, 109). During the acute phase response, liver synthesis and serum Tf levels decrease, which reduces its effectiveness in protecting LDL from oxidation (110). Multiple studies have shown that Tf levels in RA patients decrease significantly (111, 112). Studies have shown that removal of the HDL subspecies containing Tf reduces HDL’s ability to resist LDL oxidation *in vitro* (113). Because Cp-Tf has antioxidant properties, the decrease in Tf may be related to the increased consumption of Tf under RA oxidative stress and the decreased synthesis of Tf in the liver caused by the inflammatory factors IL1-βand LPS (110, 114). Therefore, it is speculated that RA inflammation and stress states further reduce the antioxidant capacity of HDL by reducing serum Tf levels.

Finally, the proteins associated with pi-HDL in RA patients also include fibrinogen (α, β, and γ chains), complement factors (C3, C9 and B), α-1-antitrypsin, Hp, Apo J, immunoglobulin heavy chain, and serpin D1 (heparin cofactor II) etc. (115). In summary, significant changes occur in pi-HDL protein particles, which are closely related to the weakening or loss of their anti-inflammatory, antioxidant, and RCT functions.

### 4.4 An increase in lipid peroxidation promotes the formation of dysfunctional HDL

The production of pi-HDL is not only attributed to changes in protein particles associated with HDL function, but also related to the lipid peroxidation of HDL. Studies have shown that oxidative stress levels in RA patients increase, and RA tends to undergo lipid peroxidation (116). The levels of lipid peroxidation markers in RA are increased, such as malondialdehyde (MDA), conjugated dienes (CD), hydroperoxides (LOOH), carbonyl proteins (CP) and so on (117). Lipid peroxides may interfere with the antioxidant, anti-inflammatory, and cholesterol receptor activity of HDL (118, 119). For example, HDL malondialdehyde modification and copper-mediated HDL oxidation both lead to a decrease in the capacity of cholesterol efflux of HDL in foam cell (120, 121); intervention with 15-lipoxygenase (an enzyme that forms lipid peroxides) can reduce HDL cholesterol receptors and anti-inflammatory activity (118, 119). Dysfunctional HDL at this time may promote the transfer of lipid hydroperoxides to apolipoprotein B-containing lipoproteins and promote the oxidation of VLDL and LDL (122).

The oxidation of HDL often occurs under inflammatory conditions. The levels of oxidative stress and HDL lipid peroxidation modification in RA patients increase, which is similar to what is observed in coronary heart disease patients (123). In summary, RA HDL lipid peroxidation may not only weaken the anti-inflammatory, antioxidant, and cholesterol efflux capacity of HDL, but also promote the oxidation of LDL and VLDL, exacerbating the process of atherosclerosis in RA.

### 4.5 The potential mechanisms of HDL decreasing in RA

The exact mechanism of RA HDL reduction is currently unknown. It is speculated to be related to the following processes. Firstly, the decreased synthesis of Apo A-I leads to a decrease in HDL levels. Studies have shown that the activation of inflammation (injection of endotoxin or pro-inflammatory cytokines including TNF-α and IL-1β) significantly inhibits the synthesis of Apo A-I in liver cells (67) (**Figure 3**). It is known that Apo A-I levels in RA circulation are significantly reduced. As Apo A-I is the most important constitutive protein of HDL, the decrease in its level can cause the synthesis process of HDL to be blocked. Secondly, the increased Apo A-I decomposition leads to a decreased level of HDL. During the acute phase of the inflammatory response, secretory phospholipase A2 reaches its highest level in plasma (66). Similarly, the serum sPLA2-IIA is elevated in RA patients and is associated with RA disease activity (124). sPLA2 can decompose the specific active site on the surface of Apo A-I, reducing HDL-c and Apo A-I (66). Thirdly, SAA replaces Apo A-I in HDL particles and accelerates HDL clearance (125). As mentioned above, under inflammatory conditions, SAA is significantly upregulated in RA circulation and replaces Apo A-I in HDL particles. HDL particles containing SAA are eliminated from circulation, which may further reduce serum HDL-c levels. Finally, under inflammatory conditions, the increase in endothelial cell lipase (ECL) promotes the clearance of HDL-c (66, 126, 127). The level of ECL increases in RA, which is associated with the process of inflammation-induced HDL level reduction (128). However, the mechanism is still not clear.

## 5 The altered subcomponent and metabolism of LDL in RA

As mentioned earlier, the overall level of LDL-C in RA tends to decrease under inflammation state. And the lower the LDL level, the higher the coronary artery calcium score in RA (129). The decrease in LDL levels in RA may be related to increased LDL oxidation, increased sub-endothelial deposition, and increased catabolism. In addition, the increase of small, dense LDL particles(sdLDL), an LDL subfraction in RA, is highly correlated with atherosclerosis.

### 5.1 The small, dense pro-atherogenic LDL particles increase in RA

LDL can be classified into 7 types based on size: LDL-I, LDL-IIa, LDL-IIb, LDL-IIIa, LDL-IIIb, LDL-IVa, and LDL-IVb. The first two types are called large buoyant LDL(IbLDL). The last five types are known as small, dense LDL (sdLDL). Research shows that sdLDL levels are significantly elevated in RA patients, and at this time the ability of LDL to specifically bind to chondroitin sulfate proteoglycan on the surface of macrophage is enhanced (130–132). The lipid profile characterized by decreased HDL-c, moderately increased TG, and elevated sdLDL is commonly referred to as atherogenic lipid phenotype and is very common in early RA (131, 133).

Studies have shown that sdLDL can promote the growth of atherosclerosis by regulating lipid metabolism, inducing inflammation, and enhancing endothelial injury (134). It is worth noting that smaller sdLDL particles are more prone to form oxidized LDL, penetrate target cells, and are not easily cleared (135–137). For example, Apo B-100 on sdLDL surface is retained in plasma for a longer period than Apo B-100 on IbLDL surface, which increases the chance of oxidation (138). In general, after combining with LDLR, LDL is cleared from the body. However, the affinity between LDLR and ApoB-100 on the surface of sdLDL molecules is low. Because of this, LDLR is incapable of recognizing sdLDL, making it easier to be absorbed by macrophages and become foam cells, which promotes atherosclerosis development and occurrence (139) (**Figure 4**). Therefore, it is speculated that the increase of RA sdLDL subgroups may accelerate the growth of atherosclerosis and increase CVD risk.

### 5.2 The possible mechanism of lipid paradox: LDL levels decrease because of increased ox-LDL and subendothelial deposition

One possible reason for the decrease in RA LDL levels is that LDL in the body is heavily oxidized and migrates to deposit under the vascular endothelium (28). Lipid deposition under the endothelium may explain the paradox of decreased LDL levels and increased CV risk in RA. Multiple studies have shown that the levels of ox-LDL and its antibodies in the blood and synovial fluid of RA patients are higher (140, 141). The carotid intima-media thickness and coronary artery calcification score in RA increase, and are positively correlated with disease activity (142–144). This suggests that, compared with the normal population, RA has a higher subclinical atherosclerotic burden. This suggests that subclinical atherosclerosis burden is higher in RA compared to the normal population.

**Figure 5:**
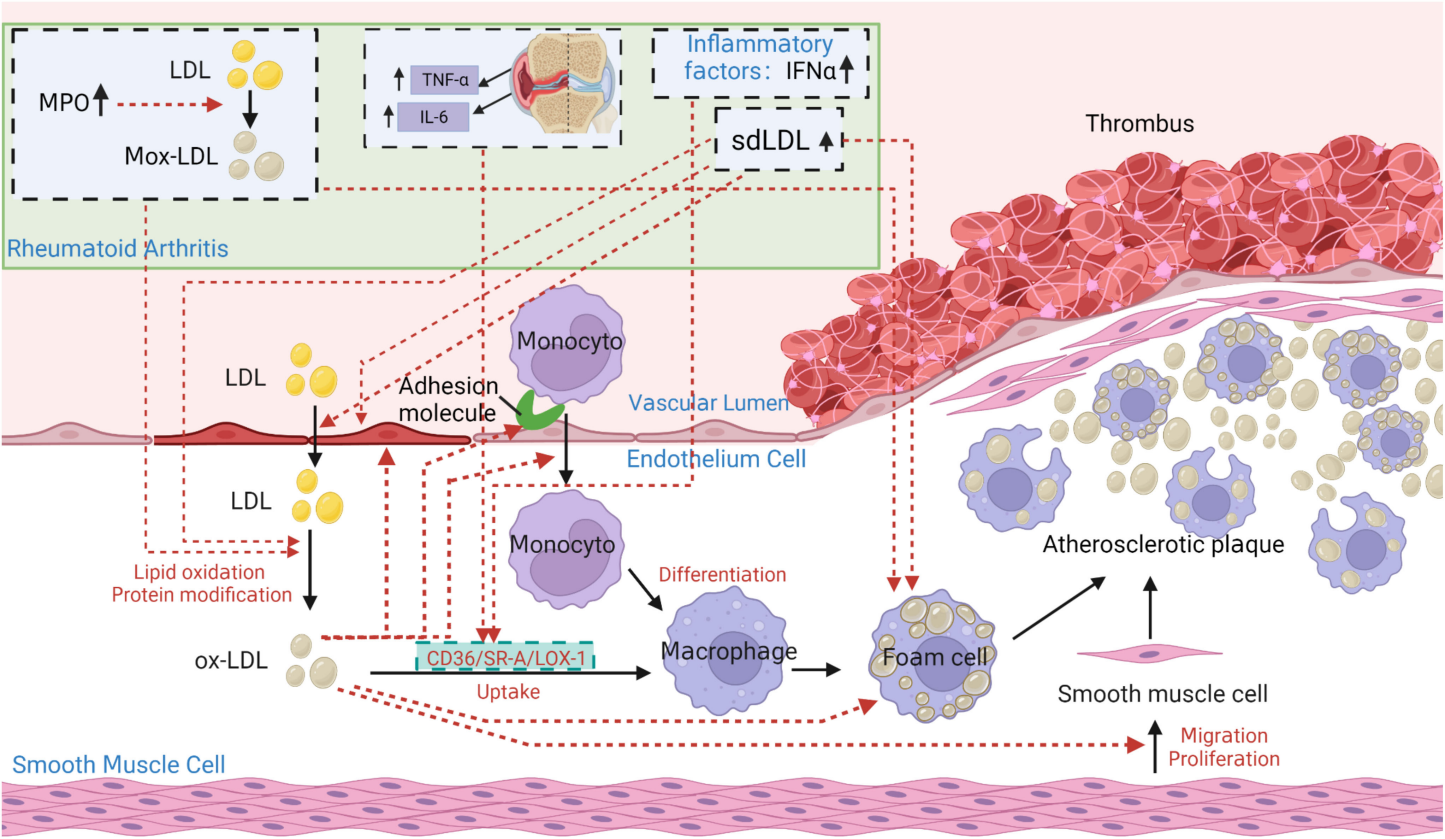
The core role of ox-LDL in the pathogenesis of atherosclerosis in patients with rheumatoid arthritis (RA). Under pathological conditions such as hypertension, hypercholesterolemia, smoking, and hyperglycemia, the vascular endothelium becomes damaged. Lipoprotein containing Apo B in plasma penetrates damaged endothelial cells into tunica intima. At the same time, the damaged endothelium expresses monocyte adhesion molecules, allowing monocytes to enter the intima and produce reactive oxygen species (ROS) to oxidize LDL to be oxidized-LDL (Ox-LDL). Ox-LDL attracts more monocytes to the site, which differentiate into macrophages. Macrophages constantly internalize ox-LDL through scavenger receptors CD36, lectin-like oxidized low-density lipoprotein receptor 1 (LOX-1), scavenger receptor class A type I/II (SR-AI/II) and class B type I(SR-BI), to accumulate as foam cells. Foam cells die, releasing their contents outside and being engulfed again by other macrophages. Ultimately, a large lesion area forms and gradually progresses into an atherosclerotic plaque. The atherosclerotic characteristics of ox-LDL can be summarized as: increasing the synthesis and secretion of adhesion molecules, chemotaxis and adhesion of monocytes, cytotoxicity to endothelial cells, enhancing foam cell formation, and increasing proliferation of smooth muscle cells. In addition, the lack of recognition of ox-LDL structure by LDLR prevents normal metabolism of LDL particles, leading to the development of atherosclerosis. In particular, under inflammatory conditions of RA, TNF, IL-6, and interferon-α (IFN-α) can enhance SR-A, LOX-1 or CD36 expression on the surface of macrophages by increasing its promoter activity in peripheral blood monocytes, thereby increasing ox-LDL uptake and enhancing foam cell formation. Elevated sdLDL in RA can promote foam cell formation and the development of atherosclerosis by regulating lipid metabolism, inducing inflammation, and enhancing endothelial injury, making itself more easily oxidized and penetrating the endothelium. Elevated Myeloperoxidase (MPO) in RA can promote LDL oxidation, leading to the formation of Mox-LDL. The clearance of Mox-LDL is reduced, exacerbating the formation of foam cells and atherosclerosis.

In the pathogenesis of atherosclerosis, Ox-LDL takes central state (145) (**Figure 5**). In particular, under inflammatory conditions of RA, interferon-α (IFN-α), IL-6, and TNF-α can enhance SR-A, lectin-like oxidized low-density lipoprotein receptor 1 (LOX-1) or CD36 expression on the surface of macrophages by increasing its promoter activity in peripheral blood monocytes, thereby increasing ox-LDL uptake and enhancing foam cell formation (35, 146). The oxidation process of LDL mainly refers to: ①under oxidative stress conditions, a large number of polyunsaturated fatty acids in LDL undergo peroxidation reactions, generating a large number of lipid oxidation products (147, 148); ②lipid oxidation products and locally produced ROS covalently modify Apo B to generate a large number of aldehyde complexes, such as MDA, 4-hydroxynonenal, and acrolein etc. (147–150). The increase in Ox-LDL in RA patients may be related not only indirectly to the decreased antioxidant capacity of HDL in RA, but also to the increase in proteins that can directly increase LDL oxidation.

Myeloperoxidase (MPO) is the main component of neutrophil granules. Abnormal expression/release of MPO in activated neutrophils amplifies inflammation and tissue damage, which provides the bas for many disease attacks (151, 152). Studies have shown that the level of MPO activity in plasma and synovial fluid of RA patients are significantly increased, which is correlated with higher disease activity and systemic inflammation (153). MPO can target Apo B-100 in LDL, rapidly adsorb on the surface of LDL, and promote the oxidation of amino acid residues, leading to the formation of oxidized lipoproteins, usually called Mox-LDL (154). The latter is not recognized by LDLR and accumulates in macrophages, causing foam cells to form (154). Therefore, MPO promotes the development of atherosclerosis by targeting the modification of Apo B amino acid residues in LDL, promoting LDL oxidation, and exacerbating foam cell formation. MPO becomes an important predictor of RA cardiovascular risk (155). In addition, the decrease in Apo A-I levels of RA may also promote the deposition of lipids under the endothelium. As Apo A-I prevents LDL aggregation, the decrease in RA Apo A-I may promote LDL aggregation and foam cell formation (156).

### 5.3 The possible mechanism of lipid paradox: LDL levels decrease because of excessive decomposition

The lower levels of RA LDL may be related to excessive breakdown under the inflammatory state (19). In Robertson’s study, the fractional catabolic rate (FCR) of baseline LDL in RA was in the range of hyper-catabolism compared to the general population, indicating active breakdown of LDL (157). Charles-Schoeman’s study showed that the FCR of cholesterol ester in RA patients was higher than that in the control group at baseline, indicating higher catabolism of cholesterol esters (29). Through CETP, cholesterol esters can be transferred from mature HDL particles to LDL particles.Therefore, an increase in cholesterol ester FCR without an increase in cholesterol esters is expected to lead to a decrease in circulating levels of HDL-c and LDL-c.

The mechanism of decreased LDL-c levels may be that the inflammatory state increases the expression of LDLR on RA hepatocytes, leading to increased LDL clearance. Pro-inflammatory cytokines, such as TNF-α and IL-6 upregulate LDLR and SR-B1 receptors on hepatocytes, increasing the uptake of LDL by the liver, which is secreted into bile and metabolized (146, 158, 159) (**Figure 3**). There is evidence that increased LDLR expression is associated with RA atherosclerosis (159). After treatment with tocilizumab anti-IL-6, RA FCR decreased, LDLR expression decreased, and they were roughly similar to those of the general population (157, 160). This may also be one of the mechanisms for the increase in LDL levels after RA anti-inflammatory treatment.

## 6 Other lipids

Generally, the level of TC in RA is decreased, which is mainly due to a decrease of lipid particles containing TC such as HDL-c (8, 161). There is controversy over changes in TG levels of RA patients (162, 163). In the preclinical stage, early stage, and acute highly inflammatory state of RA, the level of TG increases or remains unchanged (21, 28, 117, 131, 132, 163, 164). However, in chronic highly inflammatory and cachexia state of RA, TG levels decrease (165–167). The increase in TG levels in RA is related to the excessive increase of VLDL, reduced clearance, and decreased HDL-c (12, 40, 161). The decrease in TG levels in chronic active RA is caused by a slight decrease in various lipoproteins containing TG, which may be related with the reticuloendothelial system (33). Lp(a) is an LDL particle covalently bound to Apo A. Lp(a) levels in RA patients are elevated and correlated with carotid intima-media thickness (23, 33, 161, 163, 168–170). The smaller lipid subfraction Lp(a) is considered to be more prone to atherosclerosis: it seems to have an increased ability to bind with oxidized lipids; it is more likely to be located in the vessel wall because it can bind to lysine and interact with fibrinogen; and due to increased inhibition of fibrinolysis, it appears to have a pro-thrombotic effect (171).

## 7 The impact of RA autoantibodies on blood lipids and CVD risk

Compared to inflammatory factors, RF and Anti-CCP antibody (ACPA) are more specific markers for RA patients. A large cohort study demonstrated that in the preclinical arthritis and non-arthritis groups, compared to ACPA-negative individuals, ACPA-positive individuals exhibited higher levels of TC, TC/HDL, TG, Apo B, and cardiovascular risk index, but lower levels of HDL (22, 172). In contrast, RF levels significantly decrease as TG concentrations increase (173). Multiple studies have shown that compared to the healthy control group, RF/ACCP-positive patients have a higher risk of cardiovascular events (including carotid intima-media thickness (cIMT), ischemic heart disease, atherosclerosis, etc.) (172, 174, 175). However, a study targeting 160,000 postmenopausal women with RA indicated that CVD risk is closely associated with CVD risk factors, severity of joint pain, and inflammation, but not related to being positive for anti-CCP or RF (176). According to the study by Mackey, R. H. and Myasoedova E. et al., ACCP-positive RA patients have higher levels of atherosclerosis index (AI), ESR, TNF-α, and IL-6, and lower levels of HDL, with carotid intima-media thickness (cIMT) being correlated with ACCP, CRP, TNF-α, and IL-6 (172, 177). Based on this analysis, it is likely that serum positivity (ACCP/RF) compared to serum negativity may increase the risk of CVD in RA patients through higher levels of inflammation induced by it and independent mechanisms distinct from the inflammatory pathway. This dual mechanism increases the CVD risk in RA patients. According to current research, the mechanisms through which ACCP and/or RF increase the CVD risk in RA may include: ① Increased dysfunctional HDL in ACCP-positive patients compared to serum-negative patients (178); ② Association of malondialdehyde-acetaldehyde modified low-density lipoprotein (MAA-LDL), a form of oxidized LDL, with ACCP in RA patients (179); ③ Higher levels of LOX-1 and soluble LOX-1 (sLOX-1) in RF and ACCP double-seropositive RA patients, which promote macrophage uptake of ox-LDL and macrophage formation, and are correlated with antibody titers (180). In conclusion, RF and ACPAs may be involved in the link between immune mechanisms, inflammation, and lipid metabolism changes, although the exact underlying mechanisms remain not fully understood.

## 8 The impact of RA treatment medications on blood lipids and CVD risk

RA treatment medications have varying degrees of impact on blood lipid levels and CVD risk. RA treatment medications primarily include NSAIDs, glucocorticoids (GCs), traditional synthetic disease modified anti-rheumatic drugs (csDMARDs), biosynthetic disease modified anti-rheumatic drugs (bDMARDs), and targeted synthesis of disease modified anti rheumatic drugs (tsDMARDs). The following will provide a comprehensive overview of their specific effects on blood lipids and CVD risk in RA patients, which will help understand and evaluate abnormalities in blood lipid levels and CVD risk during RA treatment. It is especially important to consider the influence of RA medications on blood lipids when combining immune-based therapy for RA with lipid intervention therapy in order to achieve optimal lipid-lowering effects and better manage RA-related CVD risk.

### 8.1 NSAIDs

NSAIDs inhibit the synthesis of prostaglandins by inhibiting cyclooxygenase (COX) enzymes, producing anti-inflammatory, analgesic, and antipyretic effects to alleviate symptoms of RA. Selective COX-2 inhibitors have a better anti-inflammatory effect in RA patients, but they can block cardioprotective prostaglandins and increase the adverse cardiovascular effects of thromboxane A2, resulting in an increased risk of CVD in the general population (increased by 35%-40%) (181–185).In contrast, COX-1 inhibitors can prevent the progression of atherosclerosis (for example, low-dose aspirin can inhibit platelet-derived thromboxane A2) (181). However, in terms of outcomes, all NSAIDs, including any NSAIDs, non-selective COX inhibitors, and COX-2 inhibitors, increase the CVD risk in RA patients (185–187). NSAIDs may increase the risk of cardiovascular events and stroke by affecting blood pressure, coagulation processes, or inducing ROS-mediated oxidative stress (188).

### 8.2 Glucocorticoids

GCs can quickly suppress inflammation in RA and slow down the radiographic progression of early-stage RA (189). GCs therapy has varying effects on blood lipids and may increase TC/TG/HDL/cholesterol levels in RA patients or have no impact (190–192). Short-term low-dose glucocorticoids may increase HDL-C levels (190). However, long-term glucocorticoid treatment can exacerbate dyslipidemia and atherosclerosis. The mechanism involves GCs blocking acyl-CoA dehydrogenase activity, promoting fatty acid synthase and acetyl-CoA carboxylase activity, and inhibiting fatty acid β-oxidation. This leads to the accumulation of fat in the liver, increasing circulating TG and VLDL levels (193). GCs use in RA patients may be dose- and duration-dependent when it comes to cardiovascular risk (194). A large retrospective study, for example, showed that RA patients who were taking <5mg/day or had a cumulative dose of <750mg did not have an increased CV risk (195). However, patients with higher daily doses, higher cumulative doses, or longer treatment durations did show an increased CV risk. For instance, RA patients receiving high-dose corticosteroids (>7.5mg/day prednisolone) had twice the risk of developing heart disease compared to non-users of corticosteroids (190). Low-dose GCs treatment (5mg prednisolone) for 24 months can lead to an approximately 35% increase in the incidence of CVD in RA patients(194). Increasing evidence suggests that there are potential risks associated with GCs use that may outweigh the benefits. It is worth noting that there is no truly safe dose of GCs, and even low doses (≤5mg/day) should be used with caution as many side effects (including infections, osteoporosis, and cardiovascular diseases) may occur after long-term treatment exceeding five years (189). Therefore, when using GCs, the balance between cardiovascular risk and benefits must be carefully weighed considering factors such as disease stage, cumulative exposure, and average daily dose. It is recommended that RA patients use the lowest possible dose of GCs therapy (for the shortest duration possible), ideally <7.5mg prednisolone or its equivalent (196).

### 8.3 csDMARDs

As the first-line medication in csDMARDs, methotrexate (MTX) effectively inhibits RA inflammation and reduces the CVD risk associated with RA. MTX can reduce overall mortality by 60% and cardiovascular-related mortality by 70% (196). It exerts a protective effect on CVD through its anti-inflammatory properties and its role in reducing arterial atherosclerosis (197). The CVD protective effects of MTX involve the following pathways: ①By inhibiting dihydrofolate reductase, MTX suppresses lymphocyte proliferation and the secretion of inflammatory cytokines such as TNF-α, IL-6, IL-1, IL-8, and CRP. It also inhibits the binding of IL-1β to IL-1βR, thereby suppressing the inflammatory response (198–201); ②MTX promotes the release of anti-inflammatory endogenous nucleosides, and adenosine acts on its receptors to upregulate ABCA1 and ABCG1 (202); ③It reverses the downregulation of ABCA1 mediated by COX-2 inhibitors (202, 203); ④MTX upregulates the generation of hydrolase 27 (HY27), which promotes the efflux of intracellular cholesterol and has anti-atherosclerotic effects (202, 203); ⑤MTX reduces the expression of adhesion molecules, enhances free radical clearance, and improves endothelial function, thereby inhibiting the recruitment of pro-inflammatory T cells and monocytes (204, 205); ⑥It inhibits the formation of malondialdehyde (MDA) and acetaldehyde (AA) adducts, which may improve lipid peroxidation levels in RA patients and promote the protective function of anti-inflammatory HDL (204). In summary, MTX plays an important role in combating arterial atherosclerosis by enhancing cholesterol transport in monocytes and macrophages, limiting foam cell formation and activation, and improving endothelial function (200). While MTX may have an impact on cholesterol metabolism, its specific effects on blood lipid levels are not yet clear. Some studies show that MTX has no significant effect on lipid profiles in RA patients, while others suggest it may increase levels of TC/HDL-c/TG (187, 206–210). Therefore, further research is needed to fully understand the effects of MTX on lipid metabolism in RA patients.

Hydroxychloroquine (HCQ) exerts immunomodulatory and anti-inflammatory effects by interfering with lysosomal activity and autophagy. HCQ can inhibit the production of IL-1, IL-6, TNF-α, and IFN-γ in monocytes *in vitro*, as well as the secretion of TNF-α, IFN-α, IL-6, and other substances by plasmacytoid dendritic cells and natural killer cells (208, 210, 211). Multiple studies have shown that HCQ can lower serum levels of LDL-C, TC, and TG, increase serum levels of HDL-C, and reduce the severity of atherosclerosis, thereby lowering the cardiovascular disease CVD risk in RA patients (204, 210, 212–216). In addition to its anti-inflammatory and lipid-improving effects, HCQ’s ability to reduce CVD risk may also be related to its ability to decrease platelet aggregation and improve insulin sensitivity (215, 217, 218). However, long-term or high-dose use of HCQ may lead to cardiac toxicity, which may be associated with its impairment of lysosomal function (219–221).

Among csDMARDs, MTX, HCQ, leflunomide, and sulfasalazine have been shown to reduce the incidence and mortality of CVD (221). However, the data supporting MTX’s ability to reduce RA-related CVD events is the most robust. Hydroxychloroquine also demonstrates good efficacy, but like other csDMARDs, further research is still needed to confirm its effects.

### 8.4 bDMARDs

It is known that TNF-α is closely associated with RA inflammation, lipid abnormalities, and the process of atherosclerosis (196, 222). TNF-α inhibitors have been shown to increase TC, LDL-C, HDL-C, and TG levels in RA patients, while reducing Apo B/Apo AI ratio and CVD risk (187, 211, 222–229). Studies have also shown that long-term treatment with TNF-α inhibitors only raises TC, HDL-C, and TG levels without affecting LDL-C levels and AI (230). This suggests that TNF-α inhibitors may have potential protective effects on lipid metabolism. The cardioprotective effects of TNF-α inhibitors may be related to the following factors: ① inhibition of systemic inflammatory signals, reducing disease activity in RA patients and improving systemic lipid metabolism; ② elevation of HDL-C levels, enhancing cardio-protection (230, 231); ③ alteration of HDL-associated protein composition, such as increased serum transferrin levels, immunoglobulin J chain levels, decreased SAA1 levels, improved HDL antioxidative and cholesterol efflux capacity, and restoration of its ability to counteract atherosclerosis development (8, 204, 230, 232, 233). In addition, studies have shown that TNF inhibitors can improve ventricular dysfunction (234). However, high-dose infliximab has been shown to worsen the condition of patients with moderate to severe chronic heart failure (235). Therefore, severe heart failure remains a contraindication for the use of TNF inhibitors in RA patients at present.

IL-6 is a major regulatory factor in the liver for the synthesis of C-reactive protein (CRP), and it is one of the most abundant cytokines expressed in the synovium of RA. Similar to TNF-α, IL-6 is upregulated in the early stages of atherosclerotic plaques in arteries. Elevated levels of IL-6 and sustained elevation of CRP in RA patients are associated with increased CVD incidence and all-cause mortality (236–238). IL-6 inhibitors can reduce RA inflammation and increase levels of LDL, TG, TC, and HDL, but are accompanied by unchanged or decreased AI and decreased CVD risk (33, 239–241). Studies have shown that compared to other inflammatory cytokine inhibitors such as TNF-α inhibitor etanercept and IL-1 inhibitor canakinumab, IL-6 inhibitors can cause higher levels of LDL (10-20%) (242). As mentioned above, in RA patients under inflammatory conditions, both TNF-α and IL-6 can upregulate the LDLR and SRB1 on liver cells, promoting the uptake and metabolism of LDL (see **Figure 3**), resulting in a high metabolic state of LDL. In one study, treatment with tocilizumab, an IL-6 inhibitor, reduced this effect and resulted in increased LDL-C levels (157). Another study showed that lipid changes after IL-6 inhibitor tocilizumab treatment were not related to disease activity or changes in inflammatory markers, suggesting that IL-6 appears to be a key driving factor for LDL changes in RA (157, 160). Tocilizumab can also downregulate LDLR on the surface of hepatocytes, indicating that adverse lipid changes (increased LDL) may be a direct hepatic effect of tocilizumab. However, TNF-α inhibitor etanercept does not have this effect. Therefore, it is speculated that the reason for the higher LDL levels caused by IL-6 inhibitors is that IL-6 inhibitors can directly regulate the level of LDLR to suppress the high metabolic state of LDL in RA. Despite the increase in LDL levels after IL-6 inhibitor treatment, the CVD risk in RA patients is not increased but rather decreased. The reasons may be: ①although IL-6 inhibitors increase LDL levels, the levels of lipoproteins that are more prone to atherosclerosis (such as oxidized LDL) do not increase (243); ②compared to TNF-α inhibitors, IL-6 inhibitors are more effective in reducing levels of pro-atherosclerotic proteins, such as SAA, sPLA2, and Lp(a) (204); ③IL-6 inhibitors can reduce pro-inflammatory components and pro-atherosclerotic proteins in HDL-C, promote levels and functions of anti-atherosclerotic HDL particles (such as improved cholesterol efflux capacity mediated by HDL) (204, 244–247); ④IL-6 inhibitors can improve endothelial function, oxidative stress, and aortic stiffness in RA patients (248–251); ⑤IL-6 inhibitors can improve insulin resistance and sensitivity, which may be beneficial for cardiovascular health (252). In conclusion, when evaluating the risk of CVD associated with IL-6 inhibitors (which may include other drugs), it is important to not only consider changes in atherosclerosis-related lipid levels but also consider the overall levels and functions of anti-atherosclerotic lipids (such as subclasses, lipoproteins, and reverse cholesterol transport) and other benefits from suppressing systemic inflammation.

IL-1, along with IL-6 and TNF-α, is a key factor in RA and atherosclerosis. IL-1 can induce the expression of adhesion molecules (such as intercellular adhesion molecule-1 (ICAM-1) and vascular cell adhesion molecule-1 (VCAM-1)) and chemokines (including monocyte chemoattractant protein-1 (MCP-1)) on endothelial cells, and promote the recruitment of inflammatory cells to the vascular system through MCP-1 (17). Enhanced expression of IL-1 itself can form a positive feedback loop, leading to the production of IL-6 and matrix metalloproteinases (MMPs): IL-6 activates the acute phase response, and certain MMPs, such as MMP1, MMP8, and MMP13, can disrupt the fibrous cap of atherosclerotic plaques, increasing susceptibility to rupture and thrombus formation (17). A study recruiting over 10,000 individuals who had experienced acute myocardial infarction (AMI) for at least one month showed that IL-1 inhibitors can reduce the risk of non-fatal myocardial infarction, non-fatal stroke, or cardiovascular death by 15%, accompanied by a decrease in IL-6 or CRP, but without affecting blood lipid levels such as LDL levels (253).

### 8.5 tsDMARD

As a type of tsDMARDs, JAK inhibitors have become a recent hot topic among rheumatologists. The function of the JAK family is to send signals from extracellular cytokines (such as IL-2~7, IFN, and growth hormones) and activate downstream cascade reactions through the JAK-STAT pathway (254). Multiple studies have shown that JAK inhibitors can increase HDL-c and LDL-c levels (or TG) in RA patients (241, 255, 256). The mechanism may be that:① JAK inhibitors can reduce the FCR, inhibit the excessive decomposition of HDL-c and LDL-c, and lead to an increase in HDL-c and LDL-c levels (29); ② JAK inhibitors can downregulate IL-6 signaling, indirectly leading to an increase in blood lipid levels(257). Although both HDL and LDL increase, the LDL/HDL ratio does not seem to be affected. Compared with <0.1 mmol/L changes for both HDL and LDL with MTX, SELECT-MONO reported an increase of 0.4 mmol/L for LDL and an increase of 0.3 mmol/L for HDL (258). Even RCT studies on filgotinib have shown a decrease in the LDL/HDL ratio within 24 weeks. This may mean that HDL increases before LDL (259–261).Due to the existence of the lipid paradox, similar to other inhibitors such as TNF-α and IL-6, JAK inhibitors may increase LDL and HDL levels, which could be a response to the decreased inflammation in RA (256).

What is the impact of lipid changes caused by JAK inhibitors on cardiovascular risk? A *post hoc* analysis of six Phase III trials of tofacitinib and two LTE trials found that the increase in HDL cholesterol after 24 weeks of tofacitinib treatment was associated with a reduced risk of future major adverse cardiovascular events (MACE) (262). Furthermore, studies have shown that JAK inhibitors can increase LCAT activity, restore normal levels of small size HDL, improve HDL function, and increase the cholesterol efflux capacity of HDL (29, 178, 263). Based on this, it is speculated that the lipid changes caused by JAK inhibitors may not worsen cardiovascular risk and may even benefit cardiovascular health. However, there is a significant lag between lipid changes and cardiovascular disease, and lipid changes may take many years to translate into clinical outcomes (254). Therefore, further long-term studies are needed.

An open-label randomized clinical trial authorized by the FDA showed that in 4,362 RA patients aged ≥50 years with at least one additional cardiovascular risk factor, the JAK inhibitor tofacitinib increased the risk of MACE compared to anti-TNF-α therapy (264). However, this study did not have any other control groups. With respect to MACE, both TNF-α inhibitors and JAK inhibitors may be protective when compared to csDMARDs, other bDMARDs, or untreated RA patients. It should be noted that the included patients were aged ≥50 years and had one or more CV risk factors, and further research is needed to assess the impact of JAK inhibitors on MACE in patients aged ≤50 years or without cardiovascular risk factors. For now, JAK inhibitors seem to be safe to use at approved doses, with a safety profile similar to that of TNF blockade, for many RA patients, especially young people and elderly people without certain risk factors (such as smoking) (265).

## 9 Summary and prospects

RA has particular lipid patterns and changes in lipid composition. Quantitatively, RA shows a mild atherogenic lipid profile in the preclinical and early stages of the disease. When the inflammatory level is high or inflammation recurs, RA has an extremely similar lipid profile to other inflammatory and infectious diseases: the decreased levels of LDL-c, HDL-c, and Apo A-I (266). A decrease in HDL levels may be related to decreased synthesis and increased degradation of HDL under inflammatory conditions, with mechanisms possibly involving Apo A-I, sPLA2, SAA, and ECL. A decrease in LDL levels is associated with increased LDL oxidation, increased endothelial deposition, and increased degradation. Increased endothelial deposition and degradation of LDL are both related to increased expression of LDL-associated receptors under inflammatory conditions, leading to the migration of circulating LDL to macrophages and liver cells, causing a decrease in circulating LDL levels but an increased cardiovascular risk. Qualitatively, the formation of piHDL, decreased HDL2, and lipid peroxidation of HDL are closely related to the increased risk of CVD. The mechanism of pi-HDL formation may involve abnormal HDL functional proteins (Apo A-I, PON-1, PAF-AH, SAA, LCAT, CETP, Cp, Tf, etc.) The piHDL further promotes the atherogenic effect of ox-LDL. The increase in LDL oxidation is not only related to the decrease in HDL antioxidant function, but also to molecules such as MPO that can increase LDL oxidation. The increase of sdLDL also exacerbates the formation of atherosclerosis in RA. The changes in RA lipids are largely attributed to sustained inflammatory activity, especially the core role of IL-1, IL-6, and TNF-α.

In the state of RA inflammation, TNF-α, IL-6 and IL-1β produced locally in the joints can enter the circulation. TNF-α and IL-6 can promote LDL metabolism by increasing the expression of LDLR and SR-B1 on the surface of liver cells. TNF-α and IL-1β can inhibit the production of pro-Apo A-I particles in the liver, suppressing HDL generation. As a result, levels of both HDL and LDL in RA decrease. In addition, TNF-α, IL-6, and IFN-α can enhance SR-A, LOX-1 or CD36 expression on the surface of macrophages by increasing its promoter activity in peripheral blood monocytes, thereby increasing ox-LDL uptake and enhancing foam cell formation. Elevated sdLDL in RA can promote foam cell formation and the development of atherosclerosis by regulating lipid metabolism, inducing inflammation, and enhancing endothelial injury, making itself more easily oxidized and penetrating the endothelium. Elevated Myeloperoxidase (MPO) in RA can promote LDL oxidation, leading to the formation of Mox-LDL. The clearance of Mox-LDL is reduced, exacerbating the formation of foam cells and atherosclerosis. Overall, systemic chronic low-grade inflammation may lead to endothelial activation, dysfunctional lipid pattern, and accompanying pro-atherosclerotic and pro-thrombotic states, which are considered as the main causes of increased CVD risk in RA patients (13). Therefore, a detrimental cycle is formed: inflammatory cytokines cause lipid abnormalities, worsening atherosclerosis. This leads to further release of cytokines by endothelial cells, promoting inflammation. Therefore, actively controlling the inflammatory level of RA and stopping this vicious cycle should be the primary consideration for targeting RA lipid abnormalities.

The EULAR guidelines on cardiovascular risk management in RA emphasize that RA patients have a higher risk of CVD, and rheumatologists are responsible for managing CVD in patients with inflammatory joint diseases (267). Therefore, accurately assessing the CVD risk in RA patients and implementing appropriate proactive treatment has long-term benefits for RA patients. Firstly, the CVD risk factors in RA patients should not only consider traditional factors but also understand the significance of changes in lipid levels and function caused by inflammation, which might be better predicted by advanced lipoprotein measurements [such as changes in HDL components and cholesterol efflux capacity, or HDL Inflammatory Index(HII)] and lipid indices (such as the HDL/LDL ratio) compared to conventional lipid level testing (268). Secondly, for any persistent idiopathic synovitis, early introduction of DMARDs is recommended(2009). Early control of RA inflammation with TNF-α inhibitors and IL-6 inhibitors can help reduce CVD risk. Finally, the use of NSAIDs and GCs should align with the treatment recommendations of EULAR and the International Assessment of Spondyloarthritis Society (269), as long-term or high-dose use of these medications may increase the CVD risk in RA.

This article discusses the possible mechanisms underlying changes in RA blood lipids, which are similar to patterns seen in inflammatory diseases. Some mechanisms have not been confirmed in RA and are only speculative based on existing evidence. They need more in-depth research. This review may not encompass all the mechanisms related to changes in RA blood lipids, and more comprehensive research is expected to supplement this knowledge.

## Author contributions

JY: Conceptualization, Writing – original draft. SY: Writing – review & editing. LH: Conceptualization, Writing – original draft. XB: Conceptualization, Writing – review & editing. PS: Funding acquisition, Writing – review & editing. WL: Software, Writing – review & editing. TL: Writing – review & editing. RZ: Writing – original draft. YiH: Funding acquisition, Writing – review & editing. YaH: Funding acquisition, Validation, Writing – review & editing. KQ: Funding acquisition, Validation, Writing – review & editing. YW: Supervision, Validation, Writing – review & editing. ST: Funding acquisition, Supervision, Writing – review & editing. ZC: Conceptualization, Supervision, Visualization, Writing – review & editing.
