# Supplementary material for: Dyslipidemia in rheumatoid arthritis: the possible mechanisms
Source: Front Immunol. 2023 Oct 25;14:1254753. doi: 10.3389/fimmu.2023.1254753 (PMC10634280; doi:10.3389/fimmu.2023.1254753)
Supplement: Supplementary file 1 [file Table_1.docx]

Supplementary Material

**Dyslipidemia in rheumatoid arthritis and the possible mechanisms**

**Jiahui Yan^1^, Sisi Yang^2^, Liang Han^1^, Xin Ba^1^, Pan Shen^3^, Weiji Lin^1^, Tingting Li^1^, Ruiyuan Zhang^1^, Ying Huang^1^, Yao Huang^1^, Kai Qin^1^, Yu Wang^1^, Shenghao Tu^1^**

*** Correspondence:** Zhe Chen:zhepi2006@163.com

1 **Table 1: Summary of changes in lipid particles in rheumatoid arthritis patients**

| **References** | **Study Design** | **Phase** | **Cholesterol** | | | **TG** | **Lp(a)** | **ApoA-I** | **ApoB** | **Ratio** | |
| --- | --- | --- | --- | --- | --- | --- | --- | --- | --- | --- | --- |
|  |  |  | **TC** | **LDL-c** | **HDL-c** |  |  |  |  | **TC/HDL-c** | **LDL-c/HDL-c** |
| 18 | RA(n=79), random blood donor(n=1071) | pre-RA | ↑ |  | ↓ | ↑ |  |  | ↑ |  |  |
| 23 | RA(n=577), non-RA(n=540). | pre-RA | ↓ | ↓ | = | = |  |  |  |  |  |
| 17 | Pre-RA(n=188), non-arthritis patients(n=367). | pre-RA | ↓ |  | ↓ |  |  |  | ↓ |  |  |
| 121 | RA(n=25), healthy controls(n=22). | Early RA (durations <1 year) | = | = | ↓ | ↑ |  |  |  |  |  |
| 122 | RA(n=30), healthy controls(n=30). | Early RA ( durations <6 months) | ↑ | ↑ | ↓ | ↑ |  |  |  |  |  |
| 23 | RA(n=577), non-RA(n=540). | Within 5 years after RA diagnosis |  |  | = | = |  |  |  |  |  |
| 20 | RA(n=42) , healthy controls(n=42). | Active RA |  |  | ↓ |  | ↑ | ↓ |  | ↑ | ↑ |
| 152 | Women with RA(n=87), healthy women(n=50). | Active RA | = | = | ↓ | ↑ | ↑ |  |  | ↑ | ↑ |
| 35 | RA(n=33), controls(n=13). | Active RA(n=13), inactive RA(n=20) | = | = | ↓ | ↑ |  |  |  | ↑ |  |
| 26 | RA(n=36), controls(n=33). | Active RA. | ↓ | ↓ | ↓ |  |  | ↓ |  |  |  |
| 131 | RA(n=54), controls(n=76). | Active RA(n=27), inactive RA(n=27) | ↓ | ↓ |  |  |  |  |  |  |  |
| 158 | RA(n=28) | Active chronic RA. | ↓ | ↓ | ↓ | ↓ |  |  |  |  |  |
| 157 | RA(n=129), controls(n=1374). | Chronic RA(duration ranges 2-20 years) | ↑ | ↑ | ↓ | ↓ |  |  |  |  |  |
| 34 | RA(n=45), controls(n=45). | - | = | = | ↓ | ↑ |  | ↓ | ↑ |  |  |
| 14 | RA(n=2956), controls(n=3713). | - |  |  | ↓ |  |  |  |  |  |  |
| 28 | RA(n=2005). | - | ↓ | ↓ | = |  |  |  |  |  |  |
| 161 | RA(n=131), controls(n=200). | - |  |  |  |  | ↑ |  |  |  |  |
| 51 | RA(n=94), controls(n=79). | - | ↑ | = | = | ↑ |  | ↓ |  |  |  |
| 27 | - | - |  |  | ↓ |  | ↑ |  |  |  |  |
| 107 | RA(n=70), controls(n=40). | - | ↑ | ↑ | ↓ | ↑ |  |  |  | ↑ |  |
| 24 | RA(n=69), controls(n=65). | - | ↓ | ↓ | ↓ |  |  |  |  |  |  |
| 154 | RA(n=9), controls(n=12). | - |  |  | ↓ | ↑ |  |  |  |  |  |
| 21 | RA(n=204) | - |  | ↑ | ↓ | ↑ |  |  |  |  |  |
| 120 | RA(n=31), controls(n=28). | - | = |  | = | = |  | = | = |  |  |
| Abbreviations：RA，rheumatoid arthritis; Pre-RA, preclinical rheumatoid arthritis; TC,total cholesterol; LDL-c, low density lipoprotein cholesterol; HDL-c, high density lipoprotein cholesterol; TG, triglyceride; Lp(a), lipoprotein a; ApoA-I, apolipoprotein AI; ApoB, apolipoprotein B; GCs, glucocorticoid; DMARDs, disease-modifying antirheumatic drugs;NSAIDs, non steroidal anti-inflammatory drugs; MTX, methotrexate; =, no significant difference; ↑, increase; ↓, decrease; ↑↓, increase or decrease; -, unknown. | | | | | | | | | | | |
